# Chromosome-scale reference genome assembly of a diploid potato clone derived from an elite variety

**DOI:** 10.1093/g3journal/jkab330

**Published:** 2021-09-17

**Authors:** Ruth Freire, Marius Weisweiler, Ricardo Guerreiro, Nadia Baig, Bruno Hüttel, Evelyn Obeng-Hinneh, Juliane Renner, Stefanie Hartje, Katja Muders, Bernd Truberg, Arne Rosen, Vanessa Prigge, Julien Bruckmüller, Jens Lübeck, Benjamin Stich

**Affiliations:** 1 Institute for Quantitative Genetics and Genomics of Plants, 40225 Düsseldorf, Germany; 2 Max Planck-Genome-Centre Cologne, Max Planck Institute for Plant Breeding, 50829 Köln, Germany; 3 Böhm-Nordkartoffel Agrarproduktion GmbH & Co. OHG, 17111 Hohenmocker, Germany; 4 Nordring-Kartoffelzucht- und Vermehrungs-GmbH, 18190 Sanitz, Germany; 5 SaKa Pflanzenzucht GmbH & Co. KG, 24340 Windeby, Germany; 6 Solana Research GmbH, 24340 Windeby, Germany; 7 Cluster of Excellence on Plant Sciences, From Complex Traits towards Synthetic Modules, 40225 Düsseldorf, Germany

**Keywords:** reference sequence, elite potato variety, chromosome-scale, genome divergence, intragenomic diversity

## Abstract

Potato (*Solanum tuberosum* L.) is one of the most important crops with a worldwide production of 370 million metric tons. The objectives of this study were (1) to create a high-quality consensus sequence across the two haplotypes of a diploid clone derived from a tetraploid elite variety and assess the sequence divergence from the available potato genome assemblies, as well as among the two haplotypes; (2) to evaluate the new assembly’s usefulness for various genomic methods; and (3) to assess the performance of phasing in diploid and tetraploid clones, using linked-read sequencing technology. We used PacBio long reads coupled with 10x Genomics reads and proximity ligation scaffolding to create the dAg1_v1.0 reference genome sequence. With a final assembly size of 812 Mb, where 750 Mb are anchored to 12 chromosomes, our assembly is larger than other available potato reference sequences and high proportions of properly paired reads were observed for clones unrelated by pedigree to dAg1. Comparisons of the new dAg1_v1.0 sequence to other potato genome sequences point out the high divergence between the different potato varieties and illustrate the potential of using dAg1_v1.0 sequence in breeding applications.

## Introduction

Potato (*Solanum tuberosum* L.) was domesticated about 8000 years ago in the Andes from diploid wild potatoes and became a staple food of indigenous American communities (Spooner *et al.* 2005). Because of its high nutritional value (Jansky *et al.* 2019), the potato is nowadays one of the most important crops for humanity and its global production exceeds 370 million metric tons (FAO 2019).

The number of potato cultivars is in the thousands (FAO 2008), most of which are tetraploid (2*n* = 4× = 48), with a high level of heterozygosity and strong inbreeding depression (Zhang *et al.* 2019). With the steady rise of the human population, and growing fears of food insecurity (Beddington 2010), it is crucial to increase potato productivity. Inter alia, considerable increases are expected to be contributed by plant breeding (Lenaerts *et al.* 2019). Modern breeding tools such as genome editing (Altpeter *et al.* 2016) and genomic selection (Stich and Van Inghelandt 2018) have the potential to enhance the gain of selection in potato. However, to utilize the full potential of these tools, high-quality reference genomes of germplasm relevant to breeding are required.

The current *S. tuberosum* reference genome is that of a doubled monoploid clone from the cultivar group Phureja (Xu *et al.* 2011; Sharma *et al.* 2013; Pham *et al.* 2020). However, group Phureja has considerable genome and phenotype differences compared to the commercially established group Tuberosum of tetraploid cultivars (Xu *et al.* 2011), which makes it presumably not ideal as a reference for the latter. Furthermore, preliminary comparisons between cultivars indicated substantial sequence and structural variations (SV; Xu *et al.* 2011; Uitdewilligen *et al.* 2013), which calls for cultivar-specific genome assemblies as to optimally exploit genomic tools for potato breeding.

Assembling potato genomes is challenging because of their high levels of heterozygosity. Mixed heterozygous and homozygous regions make it difficult for algorithms to find a single unique path of overlapping reads, leading to more fragmented assemblies and a requirement of higher sequencing coverage (Pryszcz and Gabaldón 2016). If heterozygosity is very high, the alternative haplotype contigs are assumed to be separate regions of the genome, a phenomenon called undercollapsed heterozygosity (Matthews *et al.* 2018). This effect is more pronounced in tetraploid genomes, since they have more alternative haplotype versions of the same region. Long-read sequencing technologies such as PacBio (Shearman *et al.* 2020; Vollger *et al.* 2020) and Nanopore (Kuderna *et al.* 2019; Low *et al.* 2019; Kinkar *et al.* 2021) aim to overcome the problem of heterozygosity, allowing more space for overlaps during assembly (Jiao and Schneeberger 2017). Further sequencing technologies such as proximity ligation and optical mapping help resolving areas that are difficult to assemble (Field *et al.* 2020).

In recent years, potato genome assemblies of wild diploid potato relatives *S*olanum *commersonii* (Aversano *et al.* 2015) and *S*olanum *chacoense* (M6; Leisner *et al.* 2018) have become available. Exploiting the latest sequencing technological advances, Zhou *et al.* (2020) assembled the phased genome sequence of RH89-039-16 (RH89), a diploid clone derived from a cross between *S. tuberosum* dihaploid and a diploid clone, which in turn was generated from a cross between two *S. tuberosum* group Phureja hybrids (Xu *et al.* 2011). Finally, the first non-Phureja *S. tuberosum* assembly has been recently published (Solyntus_v1.1; van Lieshout *et al.* 2020). For the latter, however, the Phureja genome (DM_v4.03) has been used for reference-based scaffolding. Therefore, to our knowledge, no genome sequence of an elite variety is available nor any pure chromosome-level assembly of *S. tuberosum* group Tuberosum.

The objectives of this study were (1) to create a high-quality consensus sequence across the two haplotypes of a diploid clone derived from a tetraploid elite variety and assess the sequence divergence from the available potato genome assemblies as well as among the two haplotypes; (2) to evaluate the new assembly’s usefulness for various genomic methods; and (3) to assess the performance of phasing in diploid and tetraploid clones using linked-read sequencing technology.

## Materials and methods

### Genetic material, DNA, and RNA extraction

Three gynogenic dihaploid *S. tuberosum* clones (dAg1, dAg2, and dAg3) were created from *S. tuberosum* group Tuberosum tetraploid cv. Agria (tAg). The haploid inducer was *S. tuberosum* group Phureja IVP06-153. Besides tAg, its parental clones tPa1 and tPa2 as well as five tetraploid elite potato clones (tV1–tV5) were included in this study. DNA was extracted from the leaves of all clones according to Mayjonade *et al.* (2016). For RNA sequencing, 10 tubers of tAg were grown in a cultivation chamber set to 25°C during day (6–22 h) and 20°C during night. The light intensity was about 300 μmol/m^2^s in the leaf canopy. Samples of leaves, stolons, and flowers were harvested at 15 (leaves and stolons) and 45 (flowers) days after planting. Total RNA was extracted using RNeasy Plant MiniKit (Qiagen, Hilden, Germany) following the manufacturer’s instructions. RNA was pooled to equal concentration for the following library preparation.

### Preparation of libraries and sequencing

For all clones, 10x Genomics (10xG; Pleasanton, CA, USA) libraries were prepared (Supplementary Table S1) following the manufacturer’s recommendations, using 1 ng of DNA input, where size selection was performed before library preparation on BluePippin (SAGE Sciences, Beverly, MA, USA) with a high-pass protocol allowing a size selection start at 40 kb. The quantity and quality control of size-selected DNA were performed with Qubit (Thermo) and with a Genomic tape (Agilent TapeStation). Sequencing of 10xG libraries was performed on an Illumina (San Diego, CA, USA) HiSeq3000 in paired-end read mode.

For dAg1, SMRTbell libraries were prepared as recommended by Pacific Biosciences (Menlo Park, CA, USA, SMRTbell Template Prep Kit 1.0-SPv3), including a final size selection on Blue Pippin to remove fragments lower than 10 kb. Sequencing was performed on a PacBio Sequel I with Binding Kit 2.0 and Sequencing chemistry 2.0 for 10 h or Binding Kit 3.0 and Sequencing chemistry 3.0 for 20 h, as recommended by Pacific Biosciences.

Proximity ligation (Hi-C) data were generated for dAg1 by Dovetail (Boston, MA, USA), following the protocol of Lieberman-Aiden *et al.* (2009). A total of 129 × 10^6^ 2 × 150 bp Hi-C reads were sequenced.

Pooled RNA from leaves, stolons, and flowers was used to prepare an Iso-Seq library following manufacturer instructions. Sequencing was performed on the PacBio Sequel II using the Sequel II Sequencing Kit 2.0 chemistry. Iso-seq v3 pipeline (https://github.com/PacificBiosciences/IsoSeq) was used to generate final RNA sequencing data.

### Genome assembly

Our objective was to create one contiguous consensus assembly across the two haplotypes of dAg1 and phase the existing intragenomic variants for diploid and tetraploid clones in a second step. We have evaluated two different assembly strategies to obtain the dAg1_v1.0 genome sequence (Supplementary Figure S1), but in this manuscript, only the final assembly strategy and results are presented.

### Final assembly strategy: PacBio assembly as backbone

All PacBio reads that had ≤200× coverage, an error rate <15% after error correction, and a length ≥1000 bp were assembled with Canu v1.8 (Koren *et al.* 2017). Parallelly, the same reads were also assembled using Falcon and Falcon-unzip (Chin *et al.* 2016). To deal with the higher error rate of PacBio reads, both assemblies were polished using Pilon (v1.22; Walker *et al.* 2014) with the less error-prone 10xG linked reads, where mapping was performed with longranger align (v2.2.2) (Zheng *et al.* 2016). Furthermore, the polished Canu assembly was filtered with Purge Haplotigs (Roach *et al.* 2018) to avoid undercollapsed heterozygosity (Matthews *et al.* 2018) by discarding alternative haplotigs.

A hybrid assembly was created using quickmerge (v0.3; Chakraborty *et al.* 2016), where the polished Falcon assembly was used as reference and the polished and deduplicated Canu assembly as query. This was followed by a second round of Pilon polishing with mapped 10xG linked reads. These mapped reads were additionally used to correct misassemblies using Tigmint (Jackman *et al.* 2018) and the assembly was filtered with Purge Haplotigs. Arcs (v1.0.6; Yeo *et al.* 2018) and Links (v1.8.7; Warren *et al.* 2015) were used to scaffold contigs of the polished, corrected, deduplicated quickmerge assembly with the 10xG library 1, lowering the minimum aligned reads to 3 instead of 5 (-c 3) and using k-mers of size 20 (-k 20). Thereafter, the step was iterated with 10xG library 2. Finally, a last round of polishing with Pilon and filtering with Purge Haplotigs was performed.

### Hi-C scaffolding

The reads of the Hi-C library were mapped against the scaffolded hybrid assembly in two steps with different software. In the first step, we used BWA-MEM (v0.7.15; Li and Durbin 2010) for mapping and Salsa (Ghurye *et al.* 2017) for scaffolding, with misassembly correction activated (-m yes). In the second step, Juicer was used for mapping (Durand *et al.* 2016) and 3D-DNA (Dudchenko *et al.* 2017, 2018) for scaffolding. Contigs smaller than 12.5 kb were ignored during scaffolding and the repeat coverage misjoin threshold (--editor-repeat-coverage) was set to 3. The resulting contact maps were visualized using Juicebox (Durand *et al.* 2016; Dudchenko *et al.* 2017, 2018) and a final manual curation and scaffolding were performed.

### Evaluation of assemblies

A custom python script was used at all steps of the assembly to obtain several statistics, namely the N50, N90, L50, L90, number of Ns per 100 kb, as well as scaffold number, and total sequence length. Benchmarking Universal Single-Copy Orthologs (BUSCO; Simão *et al.* 2015) were used to assess gene completeness compared to the Solanaceae gene set (odb10; Kriventseva *et al.* 2019).

Whole-genome alignments of the final dAg1_v1.0 assembly and the four assemblies DM_v4.04, DM_v6.1, RH89, and Solyntus_v1.1 were performed with nucmer (-l 1000 -c 1000 -d 10) from the MUMMER package (v4.0.0beta2; Marçais *et al.* 2018). Additionally, for dAg1_v1.0 *vs* Solyntus_v1.1, a second alignment was performed using a lower minimum length of single exact matches (-l 100) and of a cluster of matches (-c 100) to visualize the alignment.

In order to evaluate our final assembly, mapping of 10xG linked reads from various diploid and tetraploid clones against our and the existing potato reference assemblies (dAg1_v1.0, DM_v4.04, DM_v6.1, RH89, M6, and Solyntus_v1.1) was performed using longranger align. Illumina sequencing data of the diploid wild potato species (*Solanum bukasovii*, dW; Kyriakidou *et al.* 2020) were downloaded from the SRA database and mapped against the six genomes using BWA-MEM. Samtools (v1.10; Li *et al.* 2009) was used to calculate the proportion of mapped reads and properly paired reads (Thankaswamy-Kosalai *et al.* 2017).

### Gene annotation

The MAKER pipeline (Campbell *et al.* 2014) was used to annotate genes. A custom repeat library was created with Repeatmodeler (Smit *et al.* 2013) and Mite Hunter (Han and Wessler 2010) according to Campbell *et al.* (2014). Repeatmasker (Smit *et al.* 2013) was then used to mask these repeat regions in the genome. The Iso-seq RNA data generated for tAg in this project as well as published mRNA reads (SRX4882701; Caruana *et al.* 2019) from tAg, assembled into a transcriptome with Trinity (v2.11.0) (Haas *et al.* 2013), were used as EST evidences. Protein evidences were UniProt proteins of Solanum (The UniProt Consortium 2019). Snap (Korf 2004) and Augustus (Stanke *et al.* 2008) were used as gene predictors. Orthologous analysis with UniProt proteins of Solanum was done with Orthofinder (Emms and Kelly 2019).

### Iso-seq RNA analysis

High-quality RNA reads obtained with Iso-seq version 3 pipeline (https://github.com/PacificBiosciences/IsoSeq) for tAg were mapped against dAg1_v1.0, DM_v4.04, DM_v6.1, RH89, M6, and Solyntus_v1.1 genomes using Minimap2 (Li 2018). Mapped reads against dAg1_v1.0, DM_v6.1, and RH89 were then filtered for alignments with ≥99% coverage and ≥95% identity. Redundant isoforms were removed using cDNA-Cupcake pipeline (http://github.com/Magdoll/cDNA_Cupcake). Collapsed isoforms were categorized according to dAg1_v1.0, DM_v6.1, and RH89 annotations by using SQANTI3 (Tardaguila *et al.* 2018). Alternative splicing was investigated with SUPPA2 (Trincado *et al.* 2018).

### Variant calling, phasing, and annotation

The dAg1_v1.0 assembly was used as reference to call single nucleotide variants (SNV), and small insertions and deletions (indels, <50 bp) for all potato clones. The corresponding 10xG linked reads of the diploid clones dAg1, dAg2, and dAg3 were aligned with longranger wgs (v2.2.2), and phased SNV and indels were called using freebayes (v1.3.2-40; Garrison and Marth 2012). 10xG linked reads of the three tetraploid clones tAg, tPa1, and tPa2 were mapped against the dAg1_v1.0 assembly using longranger align (v2.2.2) and variants were called by freebayes. Variants of the samples dAg1, dAg2, dAg3, tAg, tPa1, and tPa2 were filtered for a minimum depth of 10. The variants of the clones were phased with whatshap polyphase (Schrinner *et al.* 2020). The allele profiles of regions for which phase information was available for the offspring (dAg1, dAg2, dAg3, and tAg) were compared with that of the respective parents (tAg, tPa1, and tPa2). The proportion of regions with correctly phased allele profiles in the offspring compared to the allele profiles of the parental clones was calculated.

Sorting Intolerant From Tolerant 4G (SIFT4G, v2.4) was used to annotate tolerant (score >0.05) and deleterious (score ≤0.05) variants based on the conversion of amino acid sequences (Vaser *et al.* 2016). The SIFT4G database was built using SIFT4_Create_Genomic_DB with the uniref90 database, the dAg1_v1.0 sequence, and its corresponding predicted genes and proteins. The number of genes with at least one putative deleterious variant was estimated.

Pericentromeric regions of the potato chromosomes of DM_v4.03 were determined based on the recombination rates reported for the DRH population (Manrique-Carpintero *et al.* 2016). Thereafter, we determined the pericentromeric regions in the dAg1_v1.0 sequence based on the coordinates of the whole-genome alignment between dAg1_v1.0 and DM_v4.03 using show-coords from the MUMMER package. We then used a *t*-test to examine the difference of the proportion of genes with at least one deleterious variant between ∅dAg1-3 and ∅Pa1-2 in pericentromeric to subtelomeric regions for its statistical significance. Additionally, a *t*-test was used to test for a mean difference of the proportion of genes with at least one deleterious variant, calculated in 1-Mb windows across the genome, between diploid (∅dAg1-3) and tetraploid clones (∅tPa1-2).

SV between the two haplotypes of dAg1 were identified from PacBio reads, using the CuteSV algorithm (v1.0.8; Jiang *et al.* 2020) after mapping the reads with Minimap2. Sequence divergence between the assembly sequences was estimated as the proportion of the number of bp affected by SV, where the latter was extracted from the whole-genome alignments (-l 1000 -c 1000 -d 10), including all final primary scaffolds of the four potato genomes using show-diff from the MUMMER package.

## Results and discussion

### Genome assembly

Two PacBio assemblies were created for the final assembly strategy: the first with Canu comprising 14,037 contigs and the second with Falcon comprising 2,109 contigs. The Canu assembly had a larger than expected assembly size and the BUSCO analysis indicated a high proportion of duplications, both signs of undercollapsed heterozygosity. In addition, the N50 value of the Falcon assembly (0.618 Mb) was higher than that of the Canu assembly (0.203 Mb). Consequently, the Falcon assembly was used as reference and the Canu assembly as query in creating the hybrid assembly. The resulting hybrid assembly had a reduced number of contigs (1,592) and the N50 increased to 0.865 Mb. After two rounds of 10xG scaffolding, the number of scaffolds decreased to 704 and the N50 increased to 1.656 Mb, where, for the Solanaceae gene set, a BUSCO statistic of 95% was observed.

This assembly strategy based on PacBio long reads as backbone resulted in a more contiguous assembly with a closer-to-expected assembly size and a lower number of Ns (Table 1) compared to another strategy with 10xG linked reads as backbone (Supplementary Text and Table S2). Therefore, this former assembly was used for further scaffolding using Hi-C data. The two Hi-C scaffolding approaches led to drastically increased N50 values up to 57.4 Mb and a slight decrease in BUSCO statistics. The latter phenomenon was already observed by Kadota *et al.* (2020) and might be due to misassembly over-correction and/or imperfect manual curation at the last stage.

**Table 1:** Assembly statistics of different steps of our final genome assembly strategy for dAg1

Assembly step	No. of contigs	Assembly size (Mb)	Largest contig (Mb)	N50 (Mb)	N90 (Mb)	L50	L90	Ns per 100kb	BUSCO (%)
Assembling									
Canu	14,037	1,343.9	4.559	0.203	0.035	1,643	7,787	0	95
Falcon	2,109	845.7	4.904	0.618	0.206	393	1,315	0	95
quickmerge	1,592	889.7	10.609	0.865	0.276	267	974	0	95
Arcs 1×	1,055	895.9	13.589	1.440	0.407	176	635	757	95
Arcs 2×	704	788.1	13.585	1.656	0.548	136	445	977	95
Hi-C scaffolding									
Hi-C Salsa	385	788.4	29.219	5.059	1.007	41	175	1,006	95
Hi-C 3D-DNA	12 (+614)	812.2	89.719	57.412	52.458	6	12	994	94

For details see *Materials and Methods.*

The unscaffolded minor contigs were concatenated as ChrUn (65.88 Mb), which represents 8.3% of the genome and hosts 2,124 (4.7%) of the annotated genes. When ignoring Ns and including ChrUn, the final assembly size of the dAg1_v1.0 genome was 812 Mb, which is in a similar range compared to 731, 807, and 1,674 Mb (diploid genome size) for DM_v6.1, M6, and RH89, respectively (Supplementary Table S3). Considering only the 12 final scaffolds as chromosomes, the genome size of 744 Mb is larger than in M6, Solyntus_v1.1, and DM_v6.1 (499, 716, and 731 Mb).

### Evaluation of dAg1_v1.0 genome sequence

**Figure 1:**
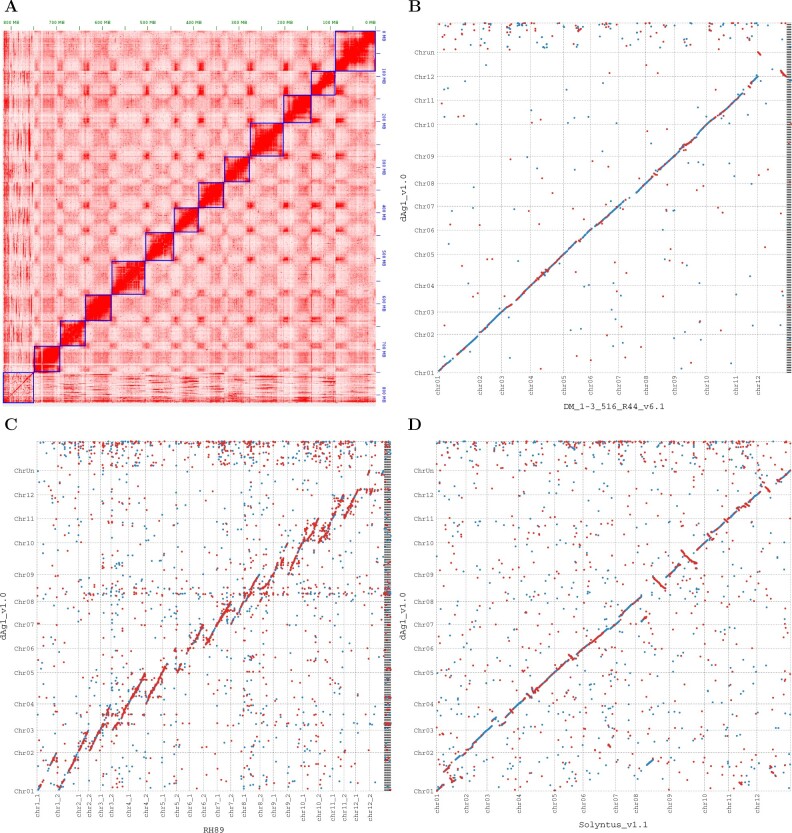
Hi-C contact map of dAg1_v1.0 sequence (A). Dot plots of whole-genome alignments of dAg1_v1.0 (vertical) *vs* DM_v6.1 (B), RH89 (C), and Solyntus_v1.1 (D) genomes (horizontal). Each dot indicates an alignment with a length of ≥1000 bp between the two genomes (≥100 bp for D). Forward and reverse alignments are represented as blue and red dots, respectively.

The visual inspection of the Hi-C contact maps of the dAg1_v1.0 sequence suggested the presence of clear contact areas between the ends of all chromosomes (Figure 1A). This has been observed earlier for plant genome (Liu and Weigel 2015; Liu *et al.* 2017) and supports the quality of our assembly. As additional quality control, especially to evaluate the successful purging of the second haplotype from the consensus assembly, we have evaluated the genome-wide distribution of the read depth. Only a few coverage spikes were observed for the final assembly (Supplementary Figure S2). A first analysis of these regions with particularly high coverage suggests that they are related to repetitive sequences. These attributes indicate that our assembly has a high quality.

Visual inspections of the dot plots of whole-genome alignments between dAg1_v1.0 *vs* DM_v6.1 and between dAg1_v1.0 *vs* RH89 (Figure 1, B and C) suggested a high level of correspondence between dAg1_v1.0 and the other two potato genomes. A reduction of the minimum length of a single exact alignment match from 1,000 to 100 bp was necessary to visualize the whole-genome alignment of dAg1_v1.0 and Solyntus_v1.1 (Figure 1D) which suggests a lower level of correspondence, which might be explained by misassemblies in the Solyntus_v1.1 genome scaffolded by DM_v4.03 (DM_v4.04 without unscaffolded contigs). This previous Phureja genome presumably included misassemblies and sequencing errors due to the limited sequencing resources available in 2011 when the genome was assembled (Pham *et al.* 2020). This explanation is supported by the observation of similar differences in the whole-genome alignment between dAg1_v1.0 and DM_v4.04 (Supplementary Figure S3) but not between dAg1_v1.0 and DM_v6.1.

The reason for larger gaps in the abovementioned whole-genome alignments might be a lower assembly quality in these regions, especially in the pericentromeric regions. The latter regions are characterized by high repeat frequencies which are difficult to assemble.

Visual inspections of the dot plots of whole-genome alignments of RH89 *vs* dAg1_v1.0 and of RH89 *vs* DM_v6.1 (Supplementary Figure S4) suggested that the RH89 and DM_v6.1 genomes are more similar than the RH89 and dAg1_v1.0 genomes. This visual impression is supported by the observation of a lower sequence divergence of ∼8.5% between RH89 and DM_v6.1 compared to ∼10.8% between RH89 and dAg_v1.0, calculated based on the sequence differences due to SV. This finding is in agreement with the RH89 pedigree, which implies a higher relatedness between RH89 and DM than between RH89 and dAg. The sequence divergence between dAg_v1.0 and DM_v6.1 and Solyntus_v1.1 was 8.2% and 8.6%, respectively.

**Figure 2:**
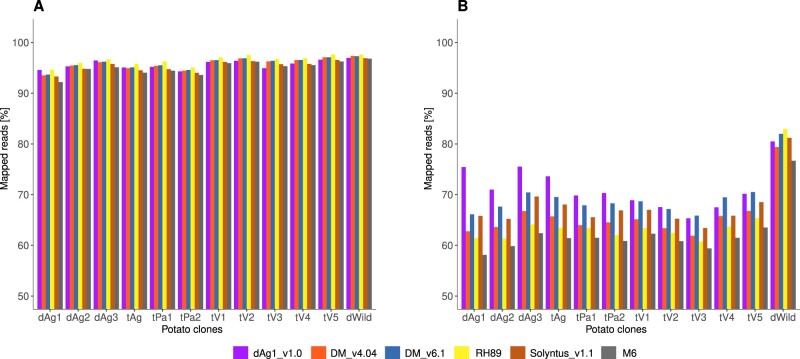
Percentage of 10xG linked reads of different potato clones mapped to different potato assemblies (A) and percentage of 10xG linked reads properly paired in mapping against different potato assemblies (B).

**Figure 3:**
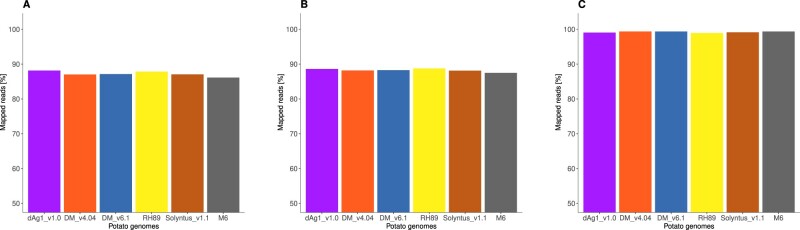
Percentage of dAg1 PacBio reads (A), tAg PacBio reads (B), and tAg high-quality Iso-seq RNA reads (C) mapped to different potato assemblies.

To assess the completeness and correctness of the dAg1_v1.0 sequence relative to other potato sequences, we mapped 10xG linked reads of various potato varieties (tV1–tV5) and one wild species (dW) to dAg1_v1.0, DM_v4.04, DM_v6.1, RH89, Solyntus_v1.1, and M6 genome sequences. The percentage of mapped reads of all examined clones was higher against *S. tuberosum* reference sequences than against M6 (Figure 2A). The proportion of mapped reads against dAg1_v1.0 was high and similar to the other *S. tuberosum* genomes. However, the proportion of properly paired reads, considered to be a more accurate quality measure (Thankaswamy-Kosalai *et al.* 2017), was on average across all examined clones the highest for the dAg1_v1.0 genome (Figure 2B). PacBio and Iso-seq reads from diploid and tetraploid Agria were also mapped against the reference assemblies and high percentages of reads were mapped in all cases (Figure 3). These observations together indicated the high completeness and especially the correctness of the dAg1_v1.0 genome assembly, which will therefore be highly useful for genome-assisted breeding applications in potato and the basis for many future research projects on diploid and tetraploid potato.

### Transcript analysis

To illustrate further the usefulness of the dAg1_v1.0 genome for research on tetraploid potatoes, a transcript analysis was performed. High-quality Iso-seq reads of tAg were mapped against dAg1_v1.0, DM_v6.1, and RH89 genomes. After collapsing the mapped reads, unique transcripts were compared with the dAg1_v1.0 annotation set as well as those published for DM_v6.1 and RH89 (Pham *et al.* 2020; Zhou *et al.* 2020). SQANTI3 analyses showed a higher ratio of transcripts associated with annotated genes compared to novel genes in dAg1_v1.0 and a higher number of annotated genes in dAg1_v1.0 compared to the other genomes (Supplementary Figure S5A). Though this finding may be biased by the fact that Iso-seq reads were used as evidences in obtaining gene models in dAg1_v1.0, the results of SQANTI3 suggest a good quality of annotation for dAg1_v1.0.

Iso-seq reads were obtained from an RNA bulk of leaves, stolons, and flowers. So a broad representation of genes should be expected. Nevertheless, some tissues, like tubers, are not present in Iso-seq reads. To assess whether dAg_v1.0 annotation presents some bias, an orthologous analysis was done between dAg_v1.0 genes and Solanum proteins from the UniProt database. Up to 90% of the tuber proteins have an orthologous in dAg_v1.0 genes, the same proportion found in RH89 and DM_v6.1. So, despite the absence of tuber Iso-seq reads, no clear bias was found in dAg_v1.0 annotated genes.

The proportion of genes with more than one isoform was greater than the number of genes with only one isoform (Supplementary Figure S6). This illustrates the importance of alternative splicing and polyadenylation (Supplementary Figure S5B). The detailed analyses of these aspects using SQANTI3 did not reveal any systematic differences (Supplementary Figure S7) compared to that described for other plants (Abdel-Ghany *et al.* 2016; Wang *et al.* 2020) and are therefore not discussed further in detail.

### Intragenomic diversity

We detected across the dAg1 genome 7,829,534 heterozygous SNV and indels which resulted in a sequence diversity between haplotypes of ∼1% (Table 2). A similar amount of heterozygous variants was identified for the two other diploid Agria clones dAg2 and dAg3. These values are in the range of what was reported previously for diploid wild species *S. commersonii* (1.49%; Aversano *et al.* 2015) and M6 (0.68%; Leisner *et al.* 2018). In addition, 32,028 SV were detected between the two dAg1 haplotypes which are in the similar range of what was reported for RH89 (Zhou *et al.* 2020). For the three tetraploid clones tAg, tPa1, and tPa2, the frequency of heterozygous variants was with 3.1%, 3.6%, and 3.3%, respectively, about thrice higher than for the diploid ones. This is due to the fact that in tetraploid clones, more variants between haplotypes can occur compared to diploid clones, as more haplotypes are present. These results are in accordance with those of Hardigan *et al*. (2017), who found a similar relation between the variant frequencies of diploid and tetraploid clones which were 1.05% for diploid landraces and 2.73% for tetraploid cultivars.

**Table 2:** Number of variants (SNV and indels) and genes with at least one deleterious variant among the haplotypes of a potato clone

Clone	Number of variants			Number of genes (del. variant)	
	Total	Heterozygous	Homozygous	Total	Homozygous
dAg1	7,829,534	7,829,534	—	13,287	—
dAg2	9,790,584	7,710,744	2,079,840	16,365	1,838
dAg3	9,461,662	7,975,910	1,485,752	16,766	1,436
tAg	25,559,532	25,495,186	64,346	26,134	25
tPa1	30,680,341	29,831,031	849,310	28,357	669
tPa2	28,666,770	27,156,995	1,509,775	27,927	1,060

The number of genes with at least one deleterious variant was assessed in our study. This number was for the diploid clones with values between 13,287 and 16,766 considerably higher than the deleterious mutations in 10,642 annotated genes described by Zhou *et al.* (2020) for the RH89 genome. This finding might be due to the usage of different approaches to detect deleterious mutations. In our study, short reads were mapped against the dAg1_v1.0 sequence, whereas Zhou *et al.* (2020) aligned the assembled chromosomes of RH89 to the DM_v4.03 sequence.

**Figure 4:**
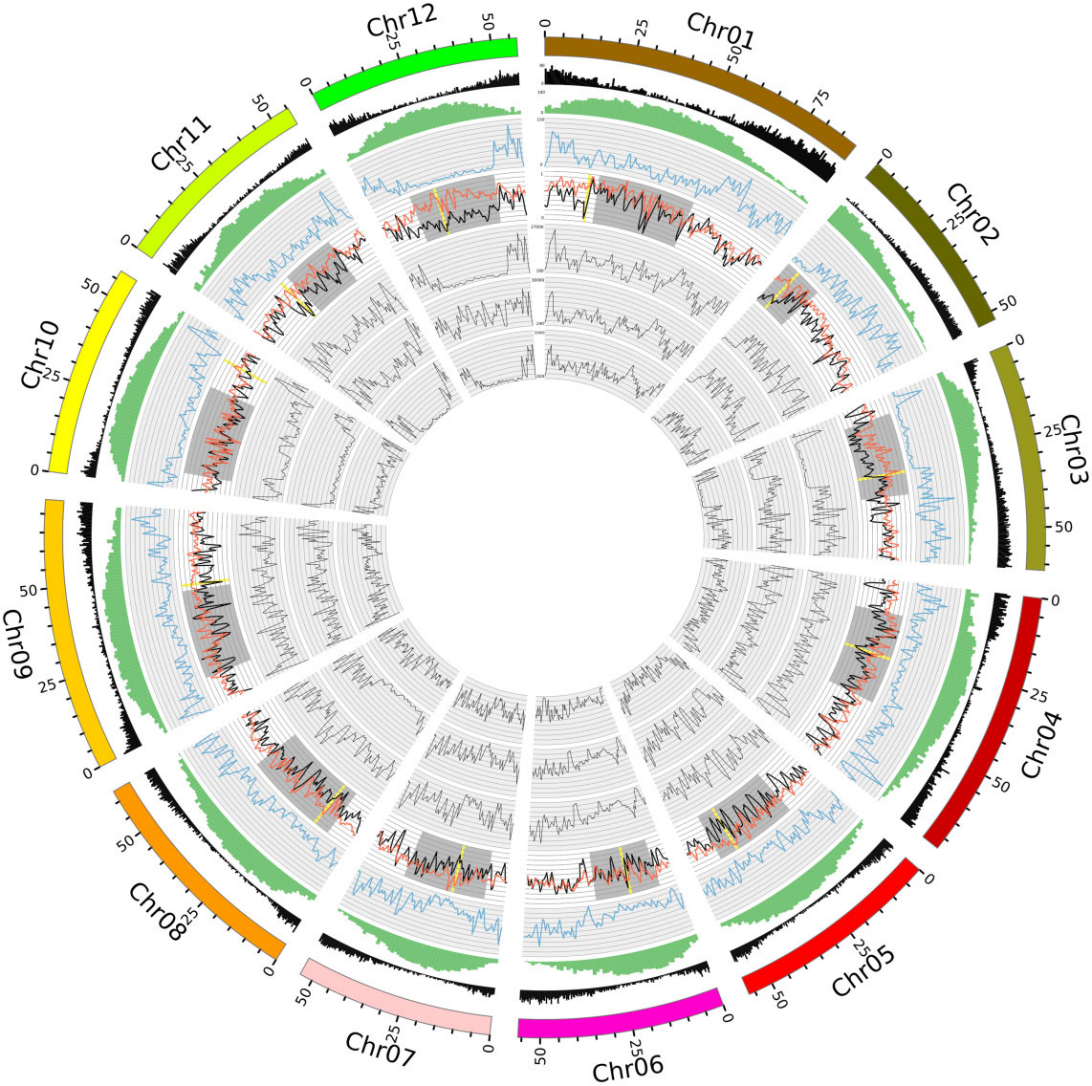
Distribution of genomic features across the potato genome. The outermost circle denotes the chromosome number and the physical position. The next inner circles report the distributions of genes (black), repeats (green) measured as percentage of masked bp, and structural variations (blue). The four most inner circles illustrate the proportion of genes with at least one deleterious variant in ∅dAg1-3 (black) and ∅tPa1-2 (orange), and heterozygous variants in dAg1, dAg2, and dAg3 in 1-Mb windows, respectively. The gray bars mark the pericentromeric regions, whereas the yellow bars mark the regions where the highest difference between the proportion of genes with at least one deleterious variant of ∅dAg1-3 and ∅tPa1-2 was identified.

The number of genes with at least one deleterious variant observed for the tetraploid clones (tPa1 and tPa2) was with values of 27,927 and 28,357 about twice as high as for the diploid clones. Hence, the proportion of genes with at least one deleterious variant in 1-Mb windows across the genome is higher for tetraploids than for diploids (Figure 4). This might be due to that in tetraploid clones deleterious alleles can be more easily masked by non-deleterious alleles due to the higher number of alleles per gene. This explanation is supported by the higher number of genes with at least one homozygous deleterious variant for diploids (dAg2: 1,838; dAg3: 1,436) than for tetraploids (tPa1: 669; tPa2: 1,060). A similar number of genes with homozygous deleterious variants (1,753) was observed by Zhou *et al.* (2020). These findings indicate the higher masking potential of deleterious alleles in tetraploids, compared to diploids. Furthermore, our observation illustrates the high efforts that will be required to breed potato as a diploid hybrid crop. This is especially true as the proportion of the number of genes with at least one deleterious variant between ∅tPa1-2 and ∅dAg1-3 was significantly (*P* < 0.001, *t*-test, sample size: 863) higher in pericentromeric regions, compared to subtelomeric regions. In the former, a purging of alleles is considerably more difficult due to the reduced recombination.

In addition to the frequency of sequence variants, the phasing of alleles is relevant to evaluate the possibilities of combining or separating alleles at neighboring loci by recombination. Recently, methods have been proposed for phasing that rely on long-read sequencing (*e.g.*, Schrinner *et al.* 2020). We have evaluated the use of linked-read sequencing for phasing the heterozygous variants for diploid and tetraploid clones. The resulting blocks of phased regions across the genomes had a median length of 116 bp for tPa2 and 6,824 bp for dAg1 (Table 3). The figures are discouraging with respect to the use of phasing information *e.g.*, in the context of genomic selection approaches (Stich and Van Inghelandt 2018). Nevertheless, these lengths are in accordance to the results of Yang *et al.* (2017) who phased the hexaploid sweet potato genome and obtained 542,361 phased regions, which covered about 30% of the genome.

**Table 3:** Q_95_, median, and Q_5_ of the block length in bp of phased variants

Clone	Q_95_ (bp)	Median (bp)	Q_5_ (bp)
dAg1	626,568	6,824	6
dAg2	341,204	267	2
dAg3	251,607	301	2
tAg	1,207	188	16
tPa1	872	131	11
tPa2	822	116	9

Despite the short block length, the phased regions of parental and offspring clones were compared to each other with respect to the present alleles. In only 2.0–2.3% of the cases, the haplotypes (*i.e.*, phased variants) observed in the three diploid clones were not observed in tAg (Table 4). For the grandparents tPa1&2, these figures were with 2.1–3.3% slightly higher but still indicating a good phasing accuracy. More than 50% of all haplotypes of tAg were also observed in the four haplotypes of the parental clones tPa1&2. It was expected that two haplotypes of tAg would occur in tPa1 and the other two in tPa2. However, an in-depth evaluation of the phasing accuracy of tetraploids using 10xG linked reads was not possible due to the short phased regions.

**Table 4:** Percentage of phased blocks for which the haplotypes of progenies occurred in 0 to multiple copies in the parental clones

Samples	dAg1	dAg2	dAg3	tAg
	0/1/2 (%)			0/1/2/3/4 (%)
tAg	2.3/9.5/ 88.2	2.0/10.8/ 87.2	2.1/9.2/ 88.7	–
tPa1	2.1/11.8/ 86.1	2.9/11.2/ 85.9	3.3/11.2/ 85.5	1.8/5.0/14.5/ 23.7/55.0
tPa2	2.1/11.4/ 86.5	2.2/10.9/ 86.9	2.9/11.2/ 85.9	1.6/4.9/14.7/ 24.9/53.9

## Conclusions

In this study, we have created a chromosome-scale consensus sequence across the two haplotypes of a diploid clone derived from a tetraploid elite potato variety. This *de novo* assembly was performed with an optimal combination of today’s sequencing technologies, comprising 10xG linked reads, PacBio long reads, and Hi-C reads. Comparisons of the new dAg1_v1.0 sequence to other potato genome assemblies pointed out the high divergence between the different potato clones and illustrated the potential of using dAg1_v1.0 sequence in breeding applications. The high amount of heterozygous SNV and indels, SV, and genes with at least one deleterious variant highlights the intragenomic diversity of the dAg1_v1.0 genome. Finally, in this study, we have shown that sequence variants of diploid potato clones could be phased using cost-efficient 10xG linked reads and the dAg1_v1.0 sequence. However, further improvements are needed to enlarge the phased regions to enable this approach in a breeding-related context.

## Data availability

Supplementary File_S1 contains Supplementary Tables S1–S3 and Figures S1–S7. Supplementary Table S1 contains statistics of the sequencing technology data used in this study, including potato clones, data type, number of reads, median, Q_5_, Q_95_, and raw coverage. Supplementary Table S2 contains assembly statistics, namely number of contigs, assembly size, largest contig, N50, N90, L50, L90, Ns per 100 kb, and percentage of BUSCO genes, for an alternative assembly strategy. Supplementary Table S3 contains statistics of the different published potato assemblies with number of scaffolds, assembly size, assembly size considering only 12 chromosomes, scaffold N50, and Ns per 100 kb for 12 chromosomes. Supplementary Figure S1 shows the pipeline of the two different assembly strategies evaluated in this study. Supplementary Figure S2 shows the read depth and the percentage of masked bp in windows across all potato chromosomes. Supplementary Figure S3 depicts the alignment dot plot of dAg1_v1.0 and DM_v4.04 genomes. Supplementary Figure S4 represents the alignment dot plot of RH89 and DM_v6.1 genomes. Supplementary Figure S5 depicts the number of transcripts assigned to annotated and novel genes and alternative splicing events. Supplementary Figure S6 shows the number of isoforms per gene. Supplementary Figure S7 indicates the frequency distribution of Full Splice Match (FSM) transcripts. The Supplementary material is available via figshare repository (doi.org/10.6084/m9.figshare.14729943). Raw sequencing data of dAg1, dAg2, dAg3, and tAg have been deposited into the NCBI Sequence Read Archive (SRA) under the accession PRJNA729250. The genome sequence of dAg1_v1.0 and the corresponding annotation files are available via figshare repository (doi.org/10.6084/m9.figshare.14604780).

## References

[jkab330-B1] Abdel-Ghany SE , Hamilton M, Jacobi JL, Ngam P, Devitt N, et al 2016. A survey of the sorghum transcriptome using single-molecule long reads. Nat Commun. 7:11706. 27339290 10.1038/ncomms11706 PMC4931028

[jkab330-B2] Altpeter F , Springer NM, Bartley LE, Blechl AE, Brutnell TP, et al 2016. Advancing crop transformation in the era of genome editing. Plant Cell. 28:1510–1520. 27335450 10.1105/tpc.16.00196 PMC4981132

[jkab330-B3] Aversano R , Contaldi F, Ercolano MR, Grosso V, Iorizzo M, et al 2015. The *Solanum commersonii* genome sequence provides insights into adaptation to stress conditions and genome evolution of wild potato relatives. Plant Cell. 27:954–968. 25873387 10.1105/tpc.114.135954 PMC4558694

[jkab330-B4] Beddington J. 2010. Food security: contributions from science to a new and greener revolution. Philos Trans R Soc Lond B Biol Sci. 365:61–71. 20008386 10.1098/rstb.2009.0201 PMC2842707

[jkab330-B5] Campbell MS , Holt C, Moore B, Yandell M. 2014. Genome annotation and curation using MAKER and MAKER-P. Curr Protoc Bioinformatics. 48:4.11.1–4.11.39. 25501943 10.1002/0471250953.bi0411s48 PMC4286374

[jkab330-B6] Caruana BM , Pembleton LW, Constable F, Rodoni B, Slater AT, et al 2019. Validation of genotyping by sequencing using transcriptomics for diversity and application of genomic selection in tetraploid potato. Front Plant Sci. 10:670. 31191581 10.3389/fpls.2019.00670 PMC6548859

[jkab330-B7] Chakraborty M , Baldwin-Brown JG, Long AD, Emerson JJ. 2016. Contiguous and accurate *de novo* assembly of metazoan genomes with modest long read coverage. Nucleic Acids Res. 44:e147. 27458204 10.1093/nar/gkw654 PMC5100563

[jkab330-B8] Chin CS , Peluso P, Sedlazeck FJ, Nattestad M, Concepcion GT, et al 2016. Phased diploid genome assembly with single-molecule real-time sequencing. Nat Methods. 13:1050–1054. 27749838 10.1038/nmeth.4035 PMC5503144

[jkab330-B9] Dudchenko O , Batra SS, Omer AD, Nyquist SK, Hoeger M, et al 2017. *De novo* assembly of the *Aedes aegypti* genome using Hi-C yields chromosome-length scaffolds. Science. 356:92–95. 28336562 10.1126/science.aal3327 PMC5635820

[jkab330-B10] Dudchenko O , Pham M, Lui C, Batra SS, Hoeger M, et al 2018. Hi-C yields chromosome-length scaffolds for a legume genome, *Trifolium subterraneum*. Preprint at: 10.1101/473553

[jkab330-B11] Durand NC , Shamim MS, Machol I, Rao SSP, Huntley MH, et al 2016. Juicer provides a one-click system for analyzing loop-resolution Hi-C experiments. Cell Syst. 3:95–98. 27467249 10.1016/j.cels.2016.07.002 PMC5846465

[jkab330-B12] Emms DM , Kelly S. 2019. OrthoFinder: phylogenetic orthology inference for comparative genomics. Genome Biol. 20:238. 31727128 10.1186/s13059-019-1832-y PMC6857279

[jkab330-B70] FAO. 2008. Statistical data. Food and Agriculture Organization of the United Nations, Rome.

[jkab330-B71] FAO. 2019. Statistical data. Food and Agriculture Organization of the United Nations, Rome.

[jkab330-B13] Field MA , Rosen BD, Dudchenko O, Chan EK, Minoche AE, et al 2020. Canfam-GSD: de novo chromosome-length genome assembly of the German Shepherd Dog (*Canis lupus familiaris*) using a combination of long reads, optical mapping, and Hi-C. GigaScience. 9:1–12. 10.1093/gigascience/giaa027 PMC7111595 32236524

[jkab330-B14] Garrison E , Marth G. 2012. Haplotype-based variant detection from short-read sequencing. arXiv:1207.3907

[jkab330-B15] Ghurye J , Pop M, Koren S, Bickhart D, Chin CS. 2017. Scaffolding of long read assemblies using long range contact information. BMC Genomics. 18:527. 28701198 10.1186/s12864-017-3879-z PMC5508778

[jkab330-B16] Haas BJ , Papanicolaou A, Yassour M, Grabherr M, Blood PD, Bowden J, et al 2013. *De novo* transcript sequence reconstruction from RNA-seq using the Trinity platform for reference generation and analysis. Nat Protoc. 8:1494–1512. 23845962 10.1038/nprot.2013.084 PMC3875132

[jkab330-B17] Han Y , Wessler SR. 2010. MITE-Hunter: a program for discovering miniature inverted-repeat transposable elements from genomic sequences. BMC Bioinformatics. 19:348. 10.1093/nar/gkq862 PMC3001096 20880995

[jkab330-B9070378] Hardigan MA, , Laimbeer FPE, , Newton L, , Crisovan E, , Hamilton JP, et al. 2017. Genome diversity of tuber-bearing Solanum uncovers complex evolutionary history and targets of domestication in the cultivated potato. Proc Natl Acad Sci U S A. 114:E9999–E10008. 10.1073/pnas.1714380114 29087343 29087343 PMC5699086

[jkab330-B18] Jackman SD , Coombe L, Chu J, Warren RL, Vandervalk BP, et al 2018. Tigmint: correcting assembly errors using linked reads from large molecules. BMC Bioinformatics. 19:393. 30367597 10.1186/s12859-018-2425-6 PMC6204047

[jkab330-B19] Jansky S , Navarre R, Bamberg J. 2019. Introduction to the special issue on the nutritional value of potato. Am J Potato Res. 96:95–97.

[jkab330-B20] Jiang T , Liu YY, Jiang Y, Li J, Gao Y, et al 2020. Long-read-based human genomic structural variation detection with cuteSV. Genome Biol. 21:189. 32746918 10.1186/s13059-020-02107-y PMC7477834

[jkab330-B21] Jiao WB , Schneeberger K. 2017. The impact of third generation genomic technologies on plant genome assembly. Curr Opin Plant Biol. 36:64–70. 28231512 10.1016/j.pbi.2017.02.002

[jkab330-B22] Kadota M , Nishimura O, Miura H, Tanaka K, Hiratani I, et al 2020. Multifaceted Hi-C benchmarking: what makes a difference in chromosome-scale genome scaffolding? Gigascience. 9:1–15. 10.1093/gigascience/giz158 PMC6952475 31919520

[jkab330-B23] Kinkar L , Gasser RB, Webster BL, Rollinson D, Littlewood DTJ, et al 2021. Nanopore sequencing resolves elusive long tandem-repeat regions in mitochondrial genomes. Int J Mol Sci. 22:1811. 33670420 10.3390/ijms22041811 PMC7918261

[jkab330-B24] Koren S , Walenz BP, Berlin K, Miller JR, Bergman NH, et al 2017. Secure because math: a deep-dive on machine learning-based monitoring. Genome Res. 27:722–736. 28298431 10.1101/gr.215087.116 PMC5411767

[jkab330-B25] Korf I. 2004. Gene finding in novel genomes. BMC Bioinformatics. 5: 59. 15144565 10.1186/1471-2105-5-59 PMC421630

[jkab330-B26] Kriventseva EV , Kuznetsov D, Tegenfeldt F, Manni M, Dias R, et al 2019. OrthoDB v10: sampling the diversity of animal, plant, fungal, protist, bacterial and viral genomes for evolutionary and functional annotations of orthologs. Nucleic Acids Res. 47:D807–D811. 30395283 10.1093/nar/gky1053 PMC6323947

[jkab330-B27] Kuderna LF , Lizano E, Julià E, Gomez-Garrido J, Serres-Armero A, et al 2019. Selective single molecule sequencing and assembly of a human Y chromosome of African origin. Nat Commun. 10:4. 30602775 10.1038/s41467-018-07885-5 PMC6315018

[jkab330-B28] Kyriakidou M , Anglin NL, Ellis D, Tai HH, Strömvik MV. 2020. Genome assembly of six polyploid potato genomes. Sci Data. 7:88. 32161269 10.1038/s41597-020-0428-4 PMC7066127

[jkab330-B29] Leisner CP , Hamilton JP, Crisovan E, Manrique-Carpintero NC, Marand AP, et al 2018. Genome sequence of M6, a diploid inbred clone of the high glycoalkaloid-producing tuber-bearing potato species *Solanum chacoense* reveals residual heterozygosity. Plant J. 94:562–570. 29405524 10.1111/tpj.13857

[jkab330-B30] Lenaerts B , Collard BC, Demont M. 2019. Review: improving global food security through accelerated plant breeding. Plant Sci. 287:110207. 31481198 10.1016/j.plantsci.2019.110207 PMC6745619

[jkab330-B31] Li H. 2018. Minimap2: pairwise alignment for nucleotide sequences. Bioinformatics. 34:3094–3100. 29750242 10.1093/bioinformatics/bty191 PMC6137996

[jkab330-B32] Li H , Durbin R. 2010. Fast and accurate long-read alignment with Burrows-Wheeler transform. Bioinformatics. 26:589–595. 20080505 10.1093/bioinformatics/btp698 PMC2828108

[jkab330-B33] Li H , Handsaker B, Wysoker A, Fennell T, Ruan J, et al; 1000 Genome Project Data Processing Subgroup. 2009. The Sequence Alignment/Map format and SAMtools. Bioinformatics. 25:2078–2079. 19505943 10.1093/bioinformatics/btp352 PMC2723002

[jkab330-B34] Lieberman-Aiden E , Berkum NLV, Williams L, Imakaev M, Ragoczy T, et al 2009. Comprehensive mapping of long-range interactions reveals folding principles of the human genome. Science. 326:289–294. 19815776 10.1126/science.1181369 PMC2858594

[jkab330-B35] Liu C , Cheng YJ, Wang JW, Weigel D. 2017. Prominent topologically associated domains differentiate global chromatin packing in rice from *Arabidopsis*. Nat Plants. 3:742–748. 28848243 10.1038/s41477-017-0005-9

[jkab330-B36] Liu C , Weigel D. 2015. Chromatin in 3D: progress and prospects for plants. Genome Biol. 16:170. 26294115 10.1186/s13059-015-0738-6 PMC4546174

[jkab330-B37] Low WY , Tearle R, Bickhart DM, Rosen BD, Kingan SB, et al 2019. Chromosome-level assembly of the water buffalo genome surpasses human and goat genomes in sequence contiguity. Nat Commun. 10:260. 30651564 10.1038/s41467-018-08260-0 PMC6335429

[jkab330-B38] Manrique-Carpintero NC , Coombs JJ, Veilleux RE, Buell CR, Douches DS. 2016. Comparative analysis of regions with distorted segregation in three diploid populations of potato. G3 (Bethesda). 6:2617–2628. 27342736 10.1534/g3.116.030031 PMC4978915

[jkab330-B39] Marçais G , Delcher AL, Phillippy AM, Coston R, Salzberg SL, et al 2018. MUMmer4: a fast and versatile genome alignment system. PLoS Comput Biol. 14:e1005944. 29373581 10.1371/journal.pcbi.1005944 PMC5802927

[jkab330-B40] Matthews BJ , Dudchenko O, Kingan SB, Koren S, Antoshechkin I, et al 2018. Improved reference genome of *Aedes aegypti* informs arbovirus vector control. Nature. 563:501–507. 30429615 10.1038/s41586-018-0692-z PMC6421076

[jkab330-B41] Mayjonade B , Gouzy J, Donnadieu C, Pouilly N, Marande W, et al 2016. Extraction of high-molecular-weight genomic DNA for long-read sequencing of single molecules. Biotechniques. 61:203–205. 27712583 10.2144/000114460

[jkab330-B42] Pham GM , Hamilton JP, Wood JC, Burke JT, Zhao H, et al 2020. Construction of a chromosome-scale long-read reference genome assembly for potato. GigaScience. 9:1–11. 10.1093/gigascience/giaa100 PMC7509475 32964225

[jkab330-B43] Pryszcz LP , Gabaldón T. 2016. Redundans: an assembly pipeline for highly heterozygous genomes. Nucleic Acids Res. 44:e113. 27131372 10.1093/nar/gkw294 PMC4937319

[jkab330-B44] Roach MJ , Schmidt SA, Borneman AR. 2018. Purge haplotigs: allelic contig reassignment for third-gen diploid genome assemblies. BMC Bioinformatics. 19:460. 30497373 10.1186/s12859-018-2485-7 PMC6267036

[jkab330-B45] Schrinner S , Mari RS, Ebler J, Rautiainen M, Seillier L, et al 2020. Haplotype threading: accurate polyploid phasing from long reads. Genome Biol. 21:252. 32951599 10.1186/s13059-020-02158-1 PMC7504856

[jkab330-B46] Sharma SK , Bolser D, de Boer J, Sønderkær M, Amoros W, et al 2013. Construction of reference chromosome-scale pseudomolecules for potato: integrating the potato genome with genetic and physical maps. G3 (Bethesda). 3:2031–2047. 24062527 10.1534/g3.113.007153 PMC3815063

[jkab330-B47] Shearman JR , Sonthirod C, Naktang C, Sangsrakru D, Yoocha T, et al 2020. Assembly of the durian chloroplast genome using long PacBio reads. Sci Rep. 10:15980. 33028920 10.1038/s41598-020-73549-4 PMC7541610

[jkab330-B48] Simão FA , Waterhouse RM, Ioannidis P, Kriventseva EV, Zdobnov EM. 2015. BUSCO: assessing genome assembly and annotation completeness with single-copy orthologs. Bioinformatics. 31:3210–3212. 26059717 10.1093/bioinformatics/btv351

[jkab330-B49] Smit A , Hubley R, Green P. 2013. RepeatMasker Open-4.0. 2013–2015. <http://www.repeatmasker.org>

[jkab330-B50] Spooner DM , McLean K, Ramsay G, Waugh R, Bryan GJ. 2005. A single domestication for potato based on multilocus amplified fragment length polymorphism genotyping. Proc Natl Acad Sci U S A. 102:14694–14699. 16203994 10.1073/pnas.0507400102 PMC1253605

[jkab330-B51] Stanke M , Diekhans M, Baertsch R, Haussler D. 2008. Using native and syntenically mapped cDNA alignments to improve *de novo* gene finding. Bioinformatics. 24:637–644. 18218656 10.1093/bioinformatics/btn013

[jkab330-B52] Stich B , Van Inghelandt D. 2018. Prospects and potential uses of genomic prediction of key performance traits in tetraploid potato. Front Plant Sci. 9:159. 29563919 10.3389/fpls.2018.00159 PMC5845909

[jkab330-B53] Tardaguila M , De La Fuente L, Marti C, Pereira C, Pardo-Palacios FJ, et al 2018. SQANTI: extensive characterization of long-read transcript sequences for quality control in full-length transcriptome identification and quantification. Genome Res. 28:369–411. 10.1101/gr.222976.117 PMC5848618 29440222

[jkab330-B54] Thankaswamy-Kosalai S , Sen P, Nookaew I. 2017. Evaluation and assessment of read-mapping by multiple next-generation sequencing aligners based on genome-wide characteristics. Genomics. 109:186–191. 28286147 10.1016/j.ygeno.2017.03.001

[jkab330-B69] The UniProt Consortium 2019. UniProt: a worldwide hub of protein knowledge. Nucleic Acids Res. 47:D506–D515. 30395287 10.1093/nar/gky1049 PMC6323992

[jkab330-B55] Trincado JL , Entizne JC, Hysenaj G, Singh B, Skalic M, et al 2018. SUPPA2: fast, accurate, and uncertainty-aware differential splicing analysis across multiple conditions. Genome Biol. 19:40. 29571299 10.1186/s13059-018-1417-1 PMC5866513

[jkab330-B56] Uitdewilligen JG , Wolters AM, D’hoop BB, Borm TJ, Visser RG, et al 2013. A next-generation sequencing method for genotyping-by-sequencing of highly heterozygous autotetraploid potato. PLoS One. 8:e62355. 23667470 10.1371/journal.pone.0062355 PMC3648547

[jkab330-B57] van Lieshout N , van der Burgt A, de Vries ME, ter Maat M, Eickholt D, Esselink D, et al 2020. Solyntus, the new highly contiguous reference genome for potato (*Solanum tuberosum*). G3 (Bethesda). 10:3489–3495. 32759330 10.1534/g3.120.401550 PMC7534448

[jkab330-B58] Vaser R , Adusumalli S, Leng SN, Sikic M, Ng PC. 2016. SIFT missense predictions for genomes. Nat Protoc. 11:1–9. 26633127 10.1038/nprot.2015.123

[jkab330-B59] Vollger MR , Logsdon GA, Audano PA, Sulovari A, Porubsky D, et al 2020. Improved assembly and variant detection of a haploid human genome using single-molecule, high-fidelity long reads. Ann Hum Genet. 84:125–140. 31711268 10.1111/ahg.12364 PMC7015760

[jkab330-B60] Walker BJ , Abeel T, Shea T, Priest M, Abouelliel A, et al 2014. Pilon: an integrated tool for comprehensive microbial variant detection and genome assembly improvement. PLoS One. 9:e112963. 25409509 10.1371/journal.pone.0112963 PMC4237348

[jkab330-B61] Wang B , Tseng E, Baybayan P, Eng K, Regulski M, et al 2020. Variant phasing and haplotypic expression from long-read sequencing in maize. Commun Biol. 3:78. 32071408 10.1038/s42003-020-0805-8 PMC7028979

[jkab330-B62] Warren RL , Yang C, Vandervalk BP, Behsaz B, Lagman A, et al 2015. LINKS: scalable, alignment-free scaffolding of draft genomes with long reads. Gigascience. 4:35. 26244089 10.1186/s13742-015-0076-3 PMC4524009

[jkab330-B63] Xu X , Pan S, Cheng S, Zhang B, Mu D, et al; Potato Genome Sequencing Consortium. 2011. Genome sequence and analysis of the tuber crop potato. Nature. 475:189–195. 21743474 10.1038/nature10158

[jkab330-B64] Yang J , Moeinzadeh MH, Hu F, Boerno S, Sun Z, et al 2017. Haplotype-resolved sweet potato genome traces back its hexaploidization history. Nat Plants. 3:696–703. 28827752 10.1038/s41477-017-0002-z

[jkab330-B65] Yeo S , Coombe L, Warren RL, Chu J, Birol I. 2018. ARCS: scaffolding genome drafts with linked reads. Bioinformatics. 34:725–731. 29069293 10.1093/bioinformatics/btx675 PMC6030987

[jkab330-B66] Zhang C , Wang P, Tang D, Yang Z, Lu F, et al 2019. The genetic basis of inbreeding depression in potato. Nat Genet. 51:374–378. 30643248 10.1038/s41588-018-0319-1

[jkab330-B67] Zheng GX , Lau BT, Schnall-Levin M, Jarosz M, Bell JM, et al 2016. Haplotyping germline and cancer genomes with high-throughput linked-read sequencing. Nat Biotechnol. 34:303–311. 26829319 10.1038/nbt.3432 PMC4786454

[jkab330-B68] Zhou Q , Tang D, Huang W, Yang Z, Zhang Y, et al 2020. Haplotype-resolved genome analyses of a heterozygous diploid potato. Nat Genet. 52:1018–1023. 32989320 10.1038/s41588-020-0699-x PMC7527274

